# On the National Vaccine Establishment

**Published:** 1815-11

**Authors:** John Walker

**Affiliations:** Director of the Royal Jennerian and London Vaccine Institutions.


*For the London Medical and Physical Journal.*

*On the National Vaccine Establishment;* by JOHN WALKER, M.D. Director of the Royal Jennerian and London Vaccine Institutions.

IN the Number for July, page 168, there is inserted the quere—How could a certain misstatement have been foisted into a solemn document, signed by the heads of two royal medical establishments, and presented to the Secretary of State? yet, in the following Number, the members of the National Vaccine Establishment do not attempt to exonerate themselves from the grave implied charge. That being the case, let the readers of the Journal find the explanation of it in line 11, *a fundo,* of page 113. It is the true one. It must also be considered as the explanation of the origin of the wondrous honours vouchsafed by the colleges to their inferior members, the physicians permitted and the surgeons *not* pure, in the late vaccination bill; hereby breaking down, or making fissures in, the sacred walls of their magic [secret] temples, the colleges of the metropolis; whence the rejected candidate, however unfairly he may think himself treated, has no appeal; and where he cannot demand an open or public examination. The present bureau of the surgeons, and those members I am acquainted with in that of Warwick-lane, are not little enough to have been the contrivers of such trumpery. But the bill having been rejected, we may not sing *Gaudeamus pueri & permissi.* We are not authorised, by the colleges, either physicians or surgeons, to certify due qualification as proposed by the rejected bill.

Had

Had the bill passed, Dr. Jenner himself, and every other physician, not licensed, *(socii etiam, permissique!)* would have been prevented from vaccinating the poor, of whatever parish, if within twelve miles of such certified licensed medical practitioner, not a member of any college.

It has long been the opprobrium of the ancient universities of St. Andrew's and Aberdeen, that they grant a diploma on the recommendation of two physicians, wherever or however these may have been dubbed. Who would have thought that the college in Warwick-lane, refusing to admit to their examinations such graduates of these ancient academies, would themselves have vouchsafed to delegate their inquisitorial powers; that the college in Lincoln's-inn Fields would have united in such departure from propriety. Let the metropolitan colleges then, themselves, be felicitated on their escape, through Lord Stanhope, from the degradation that the delegation of any, the least, of their inquisitorial powers would inevitably have brought upon them. Let them hereafter be more guarded against the ridiculous machinations of their two or three permanent officers in vaccination. While Dr. Jenner was yet in favour, during the formation of the establishment, a supernumerary permanent office was contrived, as a provision for one of his favourites, a sort of legal, not medical, practitioner; and this man, attempting to be the pioneer of the late preposterous bill to the House of Lords, has, happily, not been so successful as he was in the Court of King's Bench in arresting the progress of the propagators of the small-pox.

*Bond-court, Walbrook;*
11, *ix.* 1815.

